# Supplementary material for: HBsAg Dampened STING Associated Activation of NK Cells in HBeAg-Negative CHB Patients
Source: Int J Mol Sci. 2021 Jul 16;22(14):7643. doi: 10.3390/ijms22147643 (PMC8304816; doi:10.3390/ijms22147643)
Supplement: Supplementary file 1 [file ijms-22-07643-s001.zip › ijms-1281061-supplementary.pdf]

SUPPLEMENTARY INFORMATION

Table S1 Primers for PCR.

Table S2 Antibodies for Flow Cytometry.

Table S3. Primers for ChIP.

Figure S1. STING levels of NK cells was negatively correlated with serum HBsAg levels in CHB patients.

Figure S2. STAT3 levels was positively correlated with STING levels in NK cells of CHB patients.

Figure S3. The sequence and positions of the 5 predicted binding sites upstream of STING.

Table S1 Primers for PCR.

Name	Sequence
STING-F	GAGAGCCACCAGAGCACA
STING-R	TAGATGGACAGCAGCAACAG
IFN-α-F	TGGGCTGTGATCTGCCTCAAAC
IFN-α-R	CAGCCTTTTGGAACTGGTTGCC

Table S2 Antibodies for Flow Cytometry.

Name	Cat No.	Company
FITC anti-humanCD3	300306	Biolegend, USA
PerCP/Cy5.5 anti-human CD56	318322	Biolegend, USA
PE anti-human CD107a	328608	Biolegend, USA
Alexa Fluor® 647 anti-human/mouse Granzyme B	515406	Biolegend, USA
APC anti-human Perforin	353312	Biolegend, USA
PE anti-human IFN-γ	502509	Biolegend, USA
Alexa Fluor® 647 anti-human CD335 (NKp46)	331910	Biolegend, USA

Table S3 Primers for ChIP.

Name	Sequence
ChIP-STING-1-F	TAGATTCTTCTTGGCCTCTCTG
ChIP-STING-1-R	AAGGGAAGGTCTGACCTACATT
ChIP-STING-2-F	CGATCTCGGCTCAGTGCAACCT
ChIP-STING-2-R	AATTAGCTGGGCGTGATGGCGC
ChIP-STING-3-F	AGGCTGGTCTCAAACTTCTGGG
ChIP-STING-3-R	AAGCCTGGTGGGTTGCTCCAAA
ChIP-STING-4-F	GGCTGGTGAAATGATATACAAG
ChIP-STING-4-R	ACCTACATTGTTTGAGTGTAGG
ChIP-STING-5-F	CCACTGTGCCAGGCCTGCAATT
ChIP-STING-5-R	CAGGAAATGGCCACGCCTGTGA

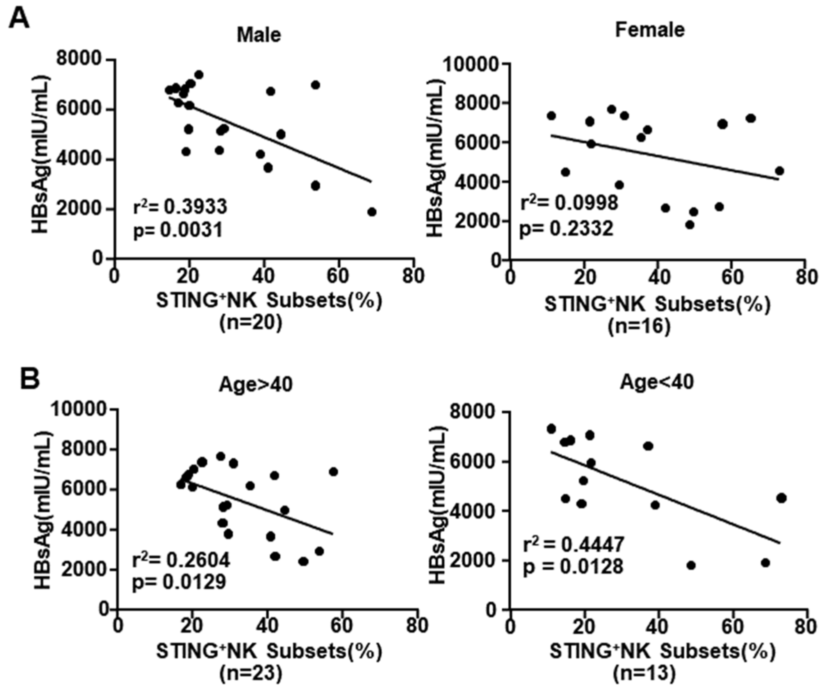

Figure S1. STING levels of NK cells was negatively correlated with serum HBsAg levels in CHB patients.

The correlation between serum HBsAg levels and STING expression levels in NK cells ($CD3^{-}CD56^{+}$) of CHB patients grouping by gender (A) and age (B). The correlation between variables was analyzed by Pearson coefficient. CHB, CHB patients; NK, natural killer.

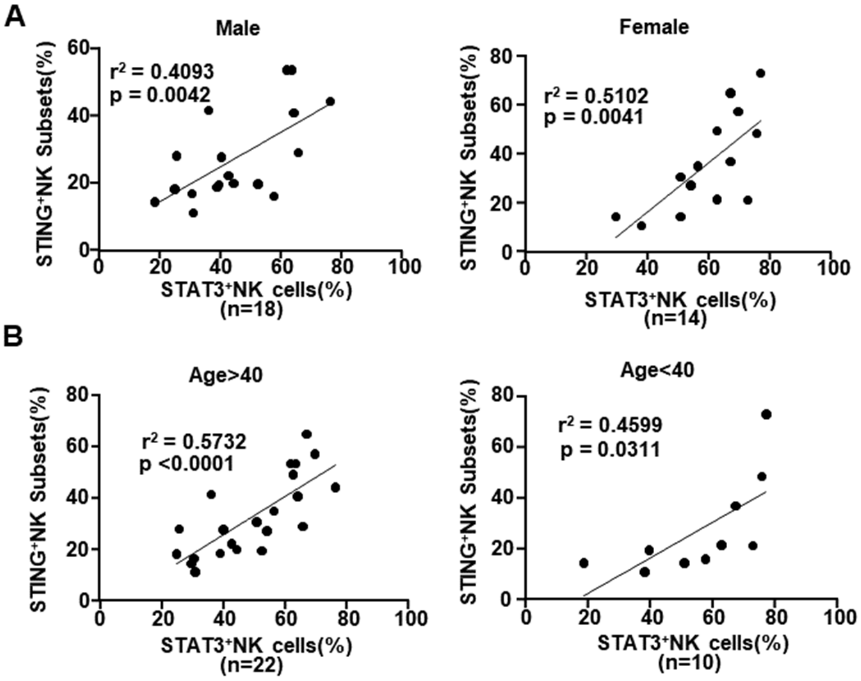

Figure S2. STAT3 levels was positively correlated with STING levels in NK cells of CHB patients.

(A-B) The correlation between STING and STAT3 expression levels in NK cells ($CD3^-$ $CD56^+$) of CHB patients grouping by gender (A) and age (B). The correlation between variables was analyzed by Pearson coefficient. CHB, CHB patients; NK, natural killer.

……Binding Site 1

-1660 TAGATTCTTC TTGGCCTCTC TGAGCGTGTG TTCCTTCTTT

-1620 CTGAGTGTGG GGCAGGGCCC TCTCTGGAAG GGGGATTTTA

-1580 TAATCTACAC TCAAACAATG TAGGTCAGAC CTTCCCTT

……Binding Site 2

-1424 AGGCTGGTCT CAAACTTCTG GGCTCAAGTG ATCCTCCTGC

-1384 CTTGGCCTCC CAAAGTGCTG GG**ATTACTGG AAT**GAAATCA

-1344 AGGCACAGAG CAAGCTGGGC TTTGGAGCAA CCCACCAGGC

-1304 TT

……Binding Site 3

-667 AGGCTGGTCT CAAACTTCTG GGCTCAAGTG ATCCTCCTGC

-627 CTTGGCCTCC CAAAGTGCTG GG**ATTACTGG AAT**GAAATCA

-587 AGGCACAGAG CAAGCTGGGC TTTGGAGCAA CCCACCAGGC

-567 TT

……Binding Site 4

-443 GGCTGGTGAA ATGATATACA AGTGAAGTGA TATATGCAAC

-403 ACTTGGCATA **ATGTCTGGAA** CAAGGTAAAC ACTTTATTAT

-363 TATTATTATT ATTATAATTT AGGTTGATGC ATGGGGATTT

-323 TATAACCTAC ACTCAAACAA TGTAGGT

……Transcription Start

AAAGCGCTGGGATTACAGGC**ATG**AG

……Binding Site 5

+3 CCACTGTGCC AGGCCTGCAA TTACTTTTGC TCCTACCTAA

+43 TATCATCCCC ACAACCGC**CT TCTGGGCAG**A_AACCGGCAGG

+83 CTCTCTTGGA GAAGTCACAG GCGTGGCCAT TTCCTGCAAA

Figure S3. The sequence and positions of the 5 predicted binding sites upstream of STING.
